# Perceived Social Influences on Women's Decisions to use Medications not Studied in Pregnancy. A Qualitative Ethical Analysis of Preexposure Prophylaxis Implementation Research in Kenya

**DOI:** 10.1177/15562646211012296

**Published:** 2021-06-16

**Authors:** Kenneth Ngure, Susan B. Trinidad, Kristin Beima-Sofie, John Kinuthia, Daniel Matemo, Grace Kimemia, Anne Njoroge, Lillian Achiro, Jillian Pintye, Nelly R. Mugo, Elizabeth A. Bukusi, Jared M. Baeten, Renee Heffron, Grace John-Stewart, Maureen C. Kelley

**Affiliations:** 1 Department of Community Health, 118985 Jomo Kenyatta University of Agriculture and Technology, Nairobi, Kenya; 2 Department of Global Health, University of Washington, Seattle, WA, USA; 3 Department of Bioethics and Humanities, University of Washington, Seattle, WA, USA; 4 Department of Research and Programs, 285569 Kenyatta National Hospital, Nairobi, Kenya; 5 Population Dynamic and Reproductive Health, 107883 African Population and Health Research Center, Nairobi, Kenya; 6 Center for Microbiology Research, 118982 Kenya Medical Research Institute Nairobi, Kenya; 7 Center for Clinical Research, Kenya Medical Research Institute Nairobi, Kenya; 8 Department of Obstetrics and Gynecology, University of Washington, Seattle, WA, USA; 9 Department of Epidemiology, University of Washington, Seattle, WA, USA; 10 Department of Medicine, University of Washington, Seattle, WA, USA; 11 Department of Pediatrics, University of Washington, Seattle, WA, USA; 12 Wellcome Centre for Ethics & Humanities and Ethox Centre, Nuffield Department of Population Health, 6396 University of Oxford, Oxford, UK

**Keywords:** research ethics, pregnancy, preexposure prophylaxis, human immunodeficiency virus prevention, partners, social influences, consent, relational autonomy

## Abstract

Implementation research ethics can be particularly challenging when pregnant women have been excluded from earlier clinical stages of research given greater uncertainty about safety and efficacy in pregnancy. The evaluation of human immunodeficiency virus (HIV) preexposure prophylaxis (PrEP) during pregnancy offered an opportunity to understand important ethical considerations and social influences shaping women's decisions to participate in the evaluation of PrEP and investigational drugs during pregnancy. We conducted interviews with women (*n* = 51), focus groups with male partners (five focus group discussions [FGDs]), interviews with health providers (*n* = 45), four FGDs with pregnant/postpartum adolescents and four FGDs with young women. Data were analyzed using thematic content analysis, including ethical aspects of the data. Our study reveals that women navigate a complex network of social influences, expectations, support, and gender roles, not only with male partners, but also with clinicians, family, and friends when making decisions about PrEP or other drugs that lack complete safety data during pregnancy.

## Background

Human immunodeficiency virus (HIV) preexposure prophylaxis (PrEP) with antiretrovirals is highly effective in preventing HIV acquisition (Baeten et al., 2012; Thigpen et al., 2012). Pregnant women in high HIV prevalence regions are at significant risk of acquiring HIV (Drake et al., 2014; Gray et al., 2005; John et al., 2006; Mugo et al., 2011; Thomson et al., 2018) and may benefit from PrEP to prevent their own HIV acquisition and vertical transmission. However, earlier clinical trials evaluating the efficacy and safety of PrEP to prevent HIV acquisition excluded pregnant women due to the investigational nature of the studies and standards for trial conduct at the time. The investigation of PrEP illustrates an ethically interesting grey area between clinical research and clinical implementation when safety data from pregnant women are unavailable and fears surrounding impact on the developing fetus are high, yet there is an urgent need for HIV prevention in maternal populations. Kenya typically follows the Council for International Organizations of Medical Sciences (CIOMS) and International Council for Harmonisation (ICH) and U.S. Federal Policy for the Protection of Human Subjects or the “Common Rule,” which include additional Subpart B protections for pregnant women, human fetuses, and neonates (CIOMS, 2016; ICH E6 (R2), 2016; USHHS, 2017). Ethical considerations related to the risk–benefit balance are assessed for each study with consent only required from the pregnant woman.

Although World Health Organization (WHO) and several country guidelines including Kenya have recommended the use of PrEP during pregnancy for women at substantial risk of HIV (NASCOP, 2017, 2018; WHO, 2017), other countries with high maternal HIV prevalence do not recommend PrEP use during pregnancy or recommend caution in light of evidence gaps (Joseph Davey et al., 2020; SANDH, 2019). In many countries with a high HIV burden, male partners play a significant role in the health-making decisions of/for women, particularly during pregnancy (Pintye et al., 2017), but the broader social dynamics and ethical considerations influencing women's decisions to participate in implementation research around HIV prevention during pregnancy are not well understood. Additionally, women's decision making during pregnancy is also influenced by health providers and families (Barnes et al., 2019; McDonald et al., 2011). The demonstration phase of PrEP research offered an opportunity to understand the factors informing the inclusion of pregnant women in implementation research in cultural contexts where making independent decisions as a pregnant woman might not be the norm. Understanding the range of social influences on women's medical decisions during pregnancy can guide discussions around offering PrEP during pregnancy in contexts like Kenya and more broadly inform how new medications are offered to pregnant women.

We conducted a cross-sectional qualitative study, Choices in Pregnancy (ChIP), within a broad context that included populations from two PrEP demonstration sites, and women seeking routine antenatal and postnatal care. The aim was to investigate the social, cultural, and ethical considerations influencing the use of PrEP—at the time an investigational drug—and other new drugs during pregnancy and lactation (Beima-Sofie et al., 2019; Ngure et al., 2017a, 2017b; Pintye et al., 2017, 2018). The focus was on the translational phase of drug development between clinical research and implementation, considering how decisions are made to include pregnant women in clinical research at that later stage. Here we report the data pertaining specifically to the social influences on Kenyan women's decisions to participate in the evaluation of PrEP and their general attitudes about taking new medications during pregnancy.

## Methods

### Setting and Study Population

The study took place in urban and periurban areas of Central and Western Kenya and data were collected from July 2015 to March 2016. The study used purposive sampling, targeting women, partners, and providers with direct experience with PrEP and those experienced with general medical decisions during pregnancy. Participants for this analysis included five cohorts: (1) pregnant or postpartum women taking PrEP, (2) nonpregnant women taking PrEP, and (3) male partners (all living with HIV) of HIV-negative women taking PrEP recruited from Thika and Kisumu sites of the Partners Demonstration Project (Baeten et al., 2016; Heffron et al., 2018). In addition, we recruited (4) health providers from Partners Demonstration Project and partnering health facilities in Thika, Kisumu, Ahero, and Mathare; and (5) PrEP unexposed women attending antenatal and postnatal clinics that were not offering PrEP in Mathare and Ahero. We relied on in-depth interviews (IDIs), focus group discussions (FGDs), and key informant interviews (KIIs) (Tables 1–5). Specifically, we conducted 21 interviews with pregnant or postpartum women who took PrEP during pregnancy and 30 interviews with nonpregnant women taking PrEP were conducted (Table 1). Sixty-eight women, including 36 nonadolescents and 32 adolescents, who were pregnant or had recently been pregnant but had not taken PrEP, participated in eight FGDs (Table 2). Additionally, five FGDs were conducted with the HIV-positive male partners of HIV-negative women taking PrEP, as follows: one FGD (*n* = 6) with partners of women who did not become pregnant and three FGDs (*n* = 22) with partners of women who did not become pregnant while taking PrEP, one FGD (*n* = 7) with a mixed group of partners (some whose partners became pregnant and some whose partners did not) (Table 3). A total of 45 health providers participated in KIIs for this portion of the study (Table 4).

**Table 1.** ChIP Participant Categories.

Population	IDI/FGD	Location	Age (years)	*N*	Partner Demonstration Project
PrEP exposed, pregnant/postpartum	IDI	Thika, Kisumu	≥18	21	Yes
PrEP exposed, no pregnancy	IDI	Thika, Kisumu	≥18	30	Yes
PrEP unexposed, pregnant/postpartum, AGYW	FGD	Mathare, Ahero	14–17	32 (4 FGDs)	No
PrEP unexposed, pregnant/postpartum	FGD	Mathare, Ahero	≥18	36 (4 FGDs)	No
Male partners	FGD	Kisumu	≥18	12 (2 FGDs)	Yes
Male partners	FGD	Thika	≥18	23 (3 FGDs)	Yes
Health providers	KII	Thika, Kisumu	≥18	45	

*Note*. ChIP = Choices in Pregnancy; IDI = in-depth interview; FGD = focus group discussion; PrEP = preexposure prophylaxis; KII = key informant interview; AGYW = Adolescent girls and young women.

**Table 2.** Female Participant Demographics.

Female FGD characteristics	Adolescent		Non-adolescent	
	*N* = 32	*N* (%) or median (IQR)	*N* = 36	*N* (%) or median (IQR)
Age	32	17 (16.5–18)	36	25 (22–29.5)
Age at first pregnancy	32	16 (15–17)	36	20.5 (19–22.5)
Number of pregnancies	32	1 (1–1.5)	36	2 (1–3)
Number of children	32	0.5 (0–1)	36	1 (0–2)
Current status	32		36	
Pregnant		19 (59)		26 (72)
Nursing		13 (41)		10 (28)
Marital status	32		36	
Married (monogamous)		22 (69)		24 (94)
Married (polygamous)		0 (0)		1 (3)
Steady boyfriend		8 (25)		0 (0)
Single		2 (6)		1 (3)
Years in relationship	24	2 (1–2)	36	3 (1–8.5)
Employment	32		36	
Housewife		12 (38)		7 (19)
Salaried		2 (6)		9 (25)
Self-employed		2 (6)		16 (44)
Unemployed		16 (50)		4 (11)
Highest level of education	32		36	
Primary		24 (75)		17 (47)
Secondary		7 (22)		18 (50)
College		1 (3)		1 (3)
Female IDI characteristics	Pregnant		Nonpregnant on PrEP	
	*N* = 21	*N* (%) or median (IQR)	*N* = 30	*N* (%) or median (IQR)
Age	21	26 (19–35)	30	33.5 (23–54)
Number of pregnancies	21	1 (0–7)	30	2 (0–5)
Number of children	21	0 (0–5)	30	2 (0–6)
Marital status (married)	21	18 (86)	30	29 (97)
Years in relationship	21	3 (0–13)	30	8.5 (0–36)
Earn an income	15	4,000 (100–80,000	21	3,000 (300–30,000)
Years in school	21	10 (2–16)	30	10 (0–16)

*Note*. IQR = interquartile range; PrEP = preexposure prophylaxis; IDI = in-depth interview.

**Table 3.** Male FGD Participant Demographics.

Characteristic	Male FGD-pregnant on PrEP		Male FGD nonpregnant on PrEP	
	*N* = 16	*N* (%) or median (IQR)	*N* = 11	*N* (%) or median (IQR)
Age		36.5 (20–53)		41 (26–65)
Number of children		0 (0–5)		1 (0–5)
Marital status (married)		15 (94%)		11 (100%)
Years in relationship^a^		2.8 (0–25)		7 (2–15)
Earn an income	16	6,000 (1,100–30,000)	11	8,000 (2,000–30,000)
Years in school		8 (0–16)		8 (4–12)

*Note*. FGD = focus group discussion; PrEP = preexposure prophylaxis; IQR = interquartile range.

^a^ Note demographics of eight male partners were missing.

**Table 4.** Health Provider Characteristics (*N* = 45).

Characteristic	*N* (%) or median (IQR)
Age	36 (25–57)
Female	30 (67)
Has children	34 (76)
Clinical training	
Nurse	7 (15.5)
Nurse counselor	8 (17.8)
Counselor	5 (11.1)
Psychologist	3 (6.7)
Clinical officer/clinician	8 (17.8)
Community health worker	4 (8.9)
Pharmacist	4 (8.9)
Other	6 (13.3)
Years of experience (total)	9 (1–34)
Years working with pregnant women	8 (1–27)

*Note*. IQR = interquartile range.

**Table 5.** Topic Guide for Male Partners, Women, and Clinicians on Considerations of Women's Autonomy (Please see Electronic Supplemental material for Detailed Interview Guides).

How did you decide to become pregnant while taking PrEP? If it was planned, did you talk about the choice to become pregnant while taking PrEP with anyone before becoming pregnant (partner, friend or relative, healthcare provider)? What about after you became pregnant—did you talk with anyone (partner, friend or relative, health provider) about staying on PrEP during your pregnancy? How do male partners view the involvement of a female partner in research during pregnancy? What kinds of concerns do they have, and how do they view the role of women in decision making? Do men expect women to obtain permission from male partners or not, and what ethical rationale is offered? How do women think about partners’ role in decisions about their own health and health during pregnancy? How do women and healthcare workers navigate social and cultural expectations when evaluating a new intervention for use during pregnancy? What, if anything, is exceptional about HIV in such decisions? Probes included comparisons to decisions involving TB or malaria treatment by comparison.

* Note*. PrEP = preexposure prophylaxis; TB = tuberculosis.

### Data Collection

ChIP was a cross-sectional qualitative study, designed to explore considerations at play for different stakeholders when deciding to offer or continue PrEP during pregnancy. To put PrEP in context, we also asked participants about medical decision making during pregnancy more broadly, using examples of tuberculosis (TB) and malaria treatments. We used semistructured guides for data collection, which were developed collaboratively between study team members based on literature reviews and professional experience in HIV prevention research and research ethics. A core set of topics was adapted for each cohort, piloted, and revised. Topic guides were translated and back-translated between English and the local dialects (Kiswahili and Luo; please see Supplemental material). Topics relevant to this analysis included: (1) the decision to become pregnant while taking PrEP, (2) the decision to continue taking PrEP during pregnancy, (3) male partner perspectives on involvement in medication use decisions during pregnancy, (4) women's perspectives on male partners’ involvement in decisions during pregnancy, (5) men's and women's concerns about using new drugs that have not been studied in pregnancy, (6) health provider perspectives on male partners’ involvement in decisions during pregnancy, (7) women's views on the role of health providers, and (8) the role of family members and friends in women's decisions. In addition to the topics above, more specific probes explored the perspectives of male partners, women, and clinicians on considerations of women's autonomy or independence and the role of others in decision making to use PrEP or new medications (Table 5). Individual interviews lasted an average of 35 min while KIIs averaged 60 min. FGDs had 6–10 participants and averaged 101 min. Interviews with women and male partners were conducted in English or the local dialects (Kiswahili or Luo) by native-speaking facilitators. Interviews with health providers were conducted in English as participants were fluent in English.

### Data Analysis

Overall, 89 transcripts from 179 participants were included in this analysis. A codebook was developed and tested by the core analysis team (KBS, JP, GT, AN, LA, and SBT), and revised as needed (MK, SBT). We used an iterative approach to team coding that included both deductive codes, derived from the research questions and topic guide, and inductive codes, emerging from the data. The iterative steps included: (1) open coding and discussion by the core team to develop a draft code list with further discussion and revisions; (2) primary coding done by (i) SBT and KBS (male partner interviews), (ii) JP, KBS, GT (IDI women), and (iii) SBT, KBS, JP, and GT (health care workers [HCWs]) with coders independently reviewing and coding clean transcripts using the final version of the codebook; (3) secondary coding, where coders exchanged transcripts, reviewing each other's application of the coding scheme. Additional codes were added and revised, and any points of disagreement were noted for discussion and possible revision with attention to disputed passages. Coding was done using ATLAS.ti, version 7. We performed a thematic content analysis using the constant comparison method to produce a description of key concepts and themes arising within and between the individual primary categories represented in the interview guides (Hsieh & Shannon, 2005). Lastly, KN, MK, and SBT conducted an ethical analysis of the data pertaining to women's decision-making autonomy in social contexts. This ethical analysis included reviewing the literature on relational agency and relational autonomy and discussing how the findings on partner and social influences might be reconciled with ethical guidance on consent and respect for women's autonomy (Burkitt, 2016; Duncan, 2015; Mackenzie & Stoljar, 2000). Our reflections included input from academic bioethics and research ethics audiences following oral presentations of this project by KN and MK (Ngure et al., 2017a, 2017b, 2018).

## Results

We spoke to men, women, and clinicians about how women make decisions to better understand the different social influences involved in making decisions to use a new medication during pregnancy. For participants involved in the Partners Demonstration Project, reflections pertained to continuation of PrEP during pregnancy. For participants outside the implementation study, these were hypothetical reflections about taking a new medication drug during pregnancy, using PrEP, TB, and malaria as examples. For many participants, the discussions led easily into general medical decisions during pregnancy. There was no notable difference in the issues raised across these different groups; so, we present these findings together but indicate when a particular attitude pertained to a research enrollment decision.

Findings revealed complex and often interconnected social influences affecting Kenyan women's decisions to consider new medications, such as PrEP, during pregnancy. Sources of influence included male partners, health providers, mothers-in-law, friends, and other family.

### Influence From Male Partners

All three groups—women, men, and health providers—reported that in the Kenyan context, male partners are involved in female partner's decisions to use PrEP or other new medications during pregnancy and have a strong influence on female partners’ decisions. We explored attitudes and experiences from these three different perspectives.

Men reported a range of views about involvement in their female partners’ decisions to take a new medication, and some men seemed internally divided about what they believed to be an appropriate role for men in this context. Most male partners reported willingness to be involved in the decision-making process in a supportive role, including accompanying the female partners to clinic visits to discuss the safety of medications during pregnancy. Among those men, some described involvement as a mutually supportive activity to do as a couple.

I will talk with her and then she goes to the doctor, we both talk with the doctor, we agree with her together with the doctor. (Male FGD, Thika)

Even her husband to be… should be informed about that research. He should be in that research…So that he can remind her when she forgets to use (study drugs) and if anything happens, he knows. (Male FGD, Thika)

Across all men in the cohort there was a consensus that men should be consulted before their female partners consider taking an investigational drug during pregnancy, but the reasons they gave for this varied. Two men used possessive language—for example, “*she is mine*” *or* “*she is my wife*”—while also speaking about the importance of support within a couple, making it difficult to describe a clear, singular reason for their belief. A few male partners believed that women needed to get a male partner's explicit permission, as opposed to the male partner being informed or consulted.

With the partner (referring to who a woman should consult) so that we speak with her first, we discuss well so that I can give her the go ahead. (Male FGD, Thika)

Considering the women's perspective, nearly all women reported that male partners play a central role in medical decisions during pregnancy and would need to be involved in choices about taking PrEP or other new medications during pregnancy. Most women believed that male partners should be informed, and all agreed that most men expect to be informed or involved in some way. Those women who decided to continue taking PrEP when they became pregnant (where the decision was not hypothetical) reported consulting their partners, who agreed, before they continued using PrEP during pregnancy; however, in some instances this consultation seemed more like permission seeking than mutual decision making. Notably, this discussion happened after discussing with the health providers. When it came to influencing on the decision, women looked to clinicians to guide their choice with their partner, but many reported that the husband could trump the decision and had the final say.

He (husband) told me because you are pregnant; you continue with taking medicine (PrEP) and I told him it was okay. (IDI PrEP experienced woman, Thika)

She can talk with her husband and maybe she can talk with the doctor if they agree with the husband, you know if the husband refuses, she cannot use them (new medications). (IDI PrEP experienced woman, Thika)

A few women believed the woman's decision required expert guidance from their health providers and viewed male partners as obstacles to be navigated. As one woman explained, some women who want to take the drug might just consult directly with their health provider, knowing that a male partner would prevent them from participating.

That is what I am telling you—the doctor where she is attending clinic (antenatal clinic). Now, that decision (to join research), the husband perhaps can refuse, and you know men are different. He might know it is this way and this way and he forbids you. But mostly the doctor is the one person …we follow very much. Because it is your health and you want, you want that health to be good so the doctor can tell them, and they feel it is good and she decides. (IDI PrEP experienced woman, Thika)

Health providers in our study confirmed what men and women reported about the strong influence of male partners on women's investigational treatment decisions in general and in the specific case of PrEP which, at this time, had not been studied in pregnancy.

Most women usually take the advice of the partner. I would just want to know whether the partner is on board because (if not) then you would be giving the drugs, and no one takes them. (KII health provider, Antenatal clinic, Mathare)

In the research context, health providers believed it is important to include male partners in women's decisions to participate in studies to support adherence and facilitate study participation. They observed that when male partners are not involved, there is poor adherence with interventions and that involving the male partner at enrollment might be the only chance to engage the male partners since many would not be available after the enrollment visit.

They [men] should [be consulted] because in our African set-up the man is the head of the house, so you find some women will get medication you’ll give them the medication but they go home and the partner does not agree to it then they tell them not to take. We have come across such cases. (KII health provider, Antenatal clinic, Ahero)

The partners should also receive the same information. At the end of the day, the support of the person taking the medication relies heavily on that person. Either from the point of whether they are going to take it or not, actually being given permission to take it or not or being influenced, yeah. If the partner is not comfortable with the medication you are taking and feels it may have an adverse effect and the person themselves is comfortable depending on the relationship. If he has more influence on over her then it will affect the adherence (KII health provider, Thika)

Health providers tended to view male partners as requiring strong involvement including providing consent before their female partners could participate in research. In the rural areas, unlike in the urban areas, providers believed many women would want their male partners involved.

When it comes to their health, again this differs by different set ups, in most of the rural set up, the women would want their partners to be part of the decision especially in the, when it comes to their health. Unlike when you go to the urban areas, the, the women sometimes would just want to be independent and make their own decisions, yeah. Whether they are going to inform the partner or not, they’ve done what they think their health needs to be done. (KII health provider, Antenatal clinic, Ahero)

Within urban areas, health providers reported that women from poor neighborhoods were more likely to defer to husbands’ wishes given greater socio-economic dependency on their male partners.

Yeah, I think it's unfortunate but in this [names poor neighborhood], most people are not empowered, they depend entirely on the husband. So, we have had people who have had to drop from the study because their husbands have said they do not want the wife to be in the study and not just mine, most of the other studies. So, I think whatever decision that we want the mother to make, I think we should incorporate the husband. I think they have a role, a very big role. (KII health provider, Antenatal clinic, Mathare)

As with some men and women in our study, some health providers acknowledged the important role that both partners play in a couple or marriage when making decisions about a child, so for decisions during pregnancy that might affect the baby, it would be unusual for a pregnant woman to make an important health decision without her partner; the reason being that it is viewed as natural to find social support within marriage.

It is good for the women to involve their partner because when they are pregnant, this is not something for one person, after all, and this child has two parents, not one, if the parent is there. Why is the mother … supposed to plan by herself? … So, they should involve their partners so that also the partners might get a better understanding of what is happening because you find most of them are much busy with work. (KII health provider, Antenatal clinic, Mathare)

### Influence From Health Providers

More than partners, women reported that health providers have a strong influence on women's decisions to take PrEP or new drugs that have not been studied in pregnancy. Overall, most women in FGDs and IDIs across all groups reported that they would follow health providers’ advice without question, because they had the expertise. A few reported that they would seek a second opinion, as illustrated by the focus group exchange below where the women were talking about how they would make important health decisions during pregnancy more generally.

P3: I also think the doctor’s advice should be final because the other people may tell you different opinions, which will not help you.

P2: The doctor’s advice is the best.

P1: According to me, what the doctor says is final and I cannot use other people’s advice.

P4: Whatever the doctor tells you is important. There is no need of consulting others elsewhere.

P5: You know some women do ignore the doctor. You may feel like that doctor did not meet your expectation and so you will ask another one.

P3: You will ask another person human being is bound to make mistakes. (Female FGD, Ahero)

Specifically, the women who continued with PrEP during pregnancy also reported that their decision was mainly informed by the health providers. The health providers counseled on the benefits of PrEP for HIV prevention during pregnancy. The health providers reported that the issue of risk was also a motivation for the women to use PrEP during pregnancy.

How to protect her from acquiring HIV, uh, first would be tests to find out whether she has no infection (HIV), and then start discussing about risk reduction, because, especially during pregnancy, chances of acquiring HIV and then the ripple effect of transmitting to the child are much higher, one you need to consider that. (KII health provider, Thika)

Yes, there are those who will say that now I want to take PrEP, and this is my situation, my partner has told you that he is taking ARVs, but I know he is not taking ARVs, he has other partners, or I suspect he has other wives or partners, and I am thinking that I am at risk. (KII health provider, Kisumu)

Men also placed significant weight on the health providers’ recommendation and information about risks to the baby when deciding whether to give permission for a female partner to take a new medication. Both men and women expressed concern about the risk of safety of the new product to the mother and the unborn baby and placed considerable weight on the health providers’ opinions and recommendations to understand these risks. If a health provider reported the possibility of side effects, most of the male partners reported that their female partners should not participate in research during pregnancy due to safety concerns.

I cannot allow mine (pregnant wife to enroll in a medication study) because if it has side effects, I will be the one in problems. Taking her to hospital, incurring the cost that I can't [pay], no. (Male FGD Thika)

When asked about whether and under what conditions women should participate in research while pregnant or take newer medications, such as PrEP, men thought potential harm to the baby outweighed potential benefits of participation, but this depended on whether risks and benefits were known and how this balance was explained by the health providers. When unknown, men in the group believed the baby's welfare should be prioritized.

She should not use (new drugs that have not been studied in pregnancy) …Because she doesn't know if, if it will harm the baby (Male FGD, Thika)

In this way, male partners and clinicians together have a strong influence on women's decisions during pregnancy—which is particularly important in the context of implementation research in the clinical settings.

### Influence From Female Family Members and Friends

Women's decision to take PrEP or other new medications during pregnancy was also influenced by other family members, especially older female family, including mothers, grandmothers, and mothers-in-law. Women's views about why female family members are consulted fell along a continuum from seeking advice to seeking permission. In some cases, the women reported seeking permission from other family members (e.g., “*She [her mother] told me to take it*.”). Others reported that although their mothers would be informed, the final decisions would primarily be influenced by what the doctor would tell them to do.

I will share this with my grandmother because I am free with her. She always asks me why they [medications] were changed, and I explain to her after that she gives me a go ahead. (Female FGD, Ahero)

The reasons women gave for involving other family members included: seeking permission, for moral support, and for social and economic support in case anything went wrong. One participant noted that in Kenyan culture some view a baby as belonging to the wider family and community after childbirth, making decisions during pregnancy family decisions. In instances where the family member was also a health provider, that person's advice would supersede that of other family members and other health providers.

I usually like to ask my mother even before I receive any kind of medication because she works in a hospital and she always advises me. I love her advice more than the person I stay with [male partner]. (Female FGD, Ahero)

I discussed with my brother since he is also a doctor so when I was afraid, I went and asked him, and he told me that that is something that is there (normally done). My brother’s advice is the one that was most important. (IDI PrEP experienced woman, Kisumu)

The first step instead of asking anybody one should ask immediate family members before making that choice because in case anything goes wrong they might ask her why she never disclosed to them; so, it is better to discuss with family members so that when you go you are in peace if in case it backfires or anything like that they will know. (Male FGD, Kisumu)

Women and health providers interviewed reported that mothers-in-law potentially have a strong influence on women's decisions to take medication during pregnancy.

It is a common thing with most of the parents from the up country they don’t like someone using drugs more so when she is pregnant, not even paracetamol. When you use them, they will start saying you are going to terminate the pregnancy. Even if you have a headache, they will refer you to their herbs, but you just must consider your stand according to the doctor’s advice. (Female FGD, Mathare)

Mothers-in-law were described as a potential barrier to pregnant women's use of PrEP or participation in research because of concerns that the use of new drugs that have not been studied in pregnancy would terminate the pregnancy. However, women also reported that their final decision would be informed by the information they would be given by the doctors.

It is obvious that mothers-in-law do refuse women to use those drugs because they say that they can terminate a pregnancy or harm the fetus. (Female FGD, Mathare)

Women also reported that friends could play a role in their decision to use PrEP, especially close female friends, and more so if they shared accommodation. These friends were described as ones who are trustworthy, who would keep issues confidential, and who would support the woman.

For example, if you have a friend who has already used them and seen the effects, she will tell you more. (Female FGD, Mathare)

There are some people who can embarrass you if you discuss with them, so you can just discus with those people you are free with. (IDI PrEP experienced woman Kisumu)

As for me I have a lady friend who has been advising me. (Female FGD Ahero)

Again, although close female friends might influence and support women's decisions during pregnancy, the final decision would be based on what the doctor advises unless the friend is also a health provider. A few women did not see any role that other family members or friends would play and would make the decision independently, but this was a minority view.

Neither an individual nor community can stop me from taking my medication when I decide to, and it is helping me. I don’t care what the family or anybody will say for as long as I know it is good and I am using it. (IDI PrEP experienced woman, Kisumu)

**Figure 1.**
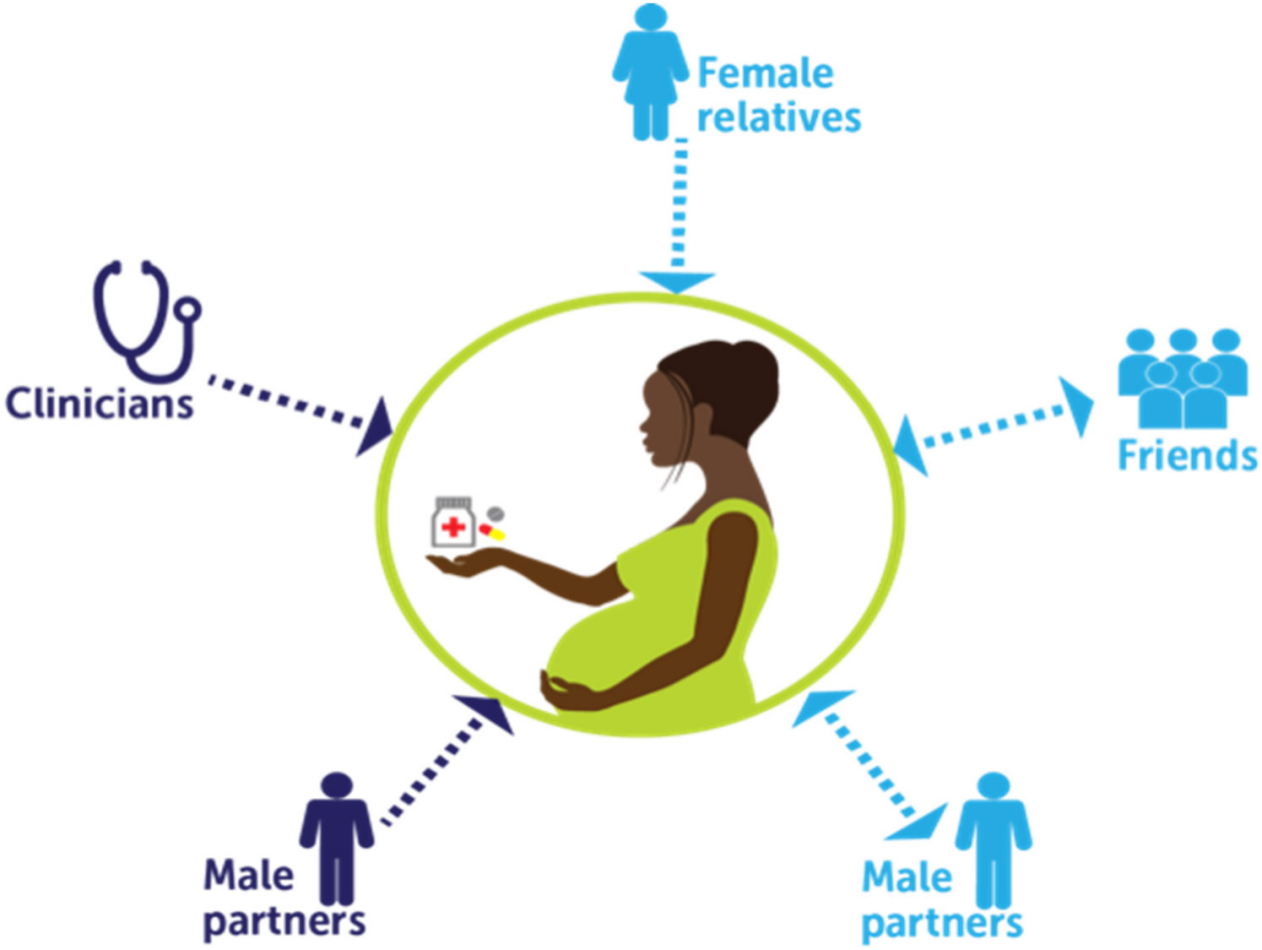
Social influences on women's decisions to use preexposure prophylaxis (PrEP) or investigational drugs during pregnancy in Kenya

*Note.* Darker color arrow represents stronger social influence relative to other people in women's lives. Direction of arrow represents direction of influence, with a single arrow representing a more directive, paternalistic interaction where advice goes largely unquestioned, and a double arrow representing a more equal, supportive conversation about a medical decision during pregnancy. Men were split between dominant/paternalistic approaches and mutually supportive decision making.

Taken together, all the sources of influence described by women, male partners, and frontline clinicians are summarized visually in Figure 1. This is a descriptive account of the multiple influences on women's treatment decisions during pregnancy, using PrEP as an example, but recognizing that participants spoke more broadly about decisions on the use of new medications in pregnancy. In the figure, the observed direction and weight of influence as described by participants are indicated. The nature of influence ranged from more directive to supportive advice in mutual conversations. The figure does not take a stance on what the role or influence of different people on women's decisions should be, or what participants thought it should be. It reflects descriptions of how things are in their experience (Figure 1).

## Discussion

In this analysis, we focused on the role of others in women's decisions when considering the use of new drugs that have not been studied in pregnancy, in particular PrEP, during pregnancy. In addition to information, women also rely upon the support and advice and navigate more authoritative influences from others, such as partners, clinicians, and mothers-in-law. As countries with a high HIV burden begin to offer PrEP and other new PrEP agents that are in development to pregnant women, it will be important to consider the various social factors involved in women's decisions during pregnancy. As we have reported elsewhere, these lessons from experiences with scaling up PrEP shed light on the wider social and ethical challenges associated with the introduction of any new medications for use by pregnant women (Ngure et al., 2017a, 2017b). Because pregnant women have often been excluded from intervention research in the interest of protecting fetal health, a decisional grey zone exists in implementation research regarding the enrolment of pregnant women. When their exclusion from intervention studies leads to their exclusion from implementation research as well, the problem is compounded: interventions that have been demonstrated to be safe and effective in nonpregnant persons may lack not only rigorous data on safety and efficacy during pregnancy and lactation, but also important insights into the features attending successful implementation during pregnancy and lactation. Thus, clinicians, women, and their partners must navigate incomplete or uncertain information when introducing new interventions into care.

Ethically, the Kenyan context illustrates the additional factors that need to be considered when planning implementation programs, such as those currently happening around PrEP, to ensure respect for women's choices within complex social relationships. While the requirement of individual informed consent persists at the heart of ethically justified research to promote the rights of an adult participant as autonomous and capable of independent decision making, many guidelines including the ICH do not adequately account for the influence of others (ICH E6 [R1], 2016). Therefore, in countries like Kenya where women typically seek social support for decisions during pregnancy ethical guidelines should include models that provide for shared decision making when desired by women. On the role of others in decision making, clinical and implementation research ethics lags behind clinical ethics and social science. The latter now recognize that values such as autonomy or agency are socially situated, and that the support of others is often critical in medical decisions within families. Work in the social sciences has demonstrated the ways in which human agency, and in this case women's agency, is in practice bounded by the views and influences of important others (Burkitt, 2016; Duncan, 2015). For example, a recent study reported that a majority of women supported the requirement for paternal consent for pregnant women's participation in clinical trials that offer the prospect of direct benefit solely to the unborn baby (Sullivan et al., 2018). What remains challenging, even with these more nuanced models of socially situated decision making, is how to distinguish between mutually valued social support for women's decisions and more problematic, paternalistic norms that view women as unable to make decisions over their health and body once they become pregnant. While a few participants, particularly the male participants, expressed this extreme view, the majority of participants saw decision making during pregnancy as a family affair.

Early work on the involvement of male partners in women's HIV testing prompted research teams and clinical teams to adopt creative strategies for navigating the expectation among many men that their permission would be sought when enrolling women in studies or programs (Ezeanolue et al., 2017). More recent work demonstrates the importance of engaging men in women's reproductive health as allies, to support improved health for women and girls (Stern et al., 2015). Our results echo what is known about the value of partner involvement but reflect a more complex network of social influences, expectations, and reinforcement of gender roles that women navigate when making decisions to use a new medication during pregnancy, and in turn, that researchers and clinicians must also recognize and devise appropriate strategies to negotiate. We found in the Kenyan context that although women had strong reasons to continue PrEP use during pregnancy to prevent HIV, especially to their unborn babies (Pintye et al., 2017), health providers and male partners reinforce a combined, dominant influence on women's treatment and research participation decisions, and that many women readily defer to partners and providers. However, men's role and influence in women's decisions varied. Not surprisingly, both women and partners reported that important decisions, like those affecting a healthy pregnancy, are made together within an intimate relationship. The few women who thought they should be able to make an independent decision, still acknowledged the pragmatic reality of needing to consult with partners, with some seeking partner permission, often along with advice or consultation with older female family members, such as a mother-in-law.

We found that family, mothers, mothers-in-law, and female friends were strongly influential and were also sought out by women. This strong family influence has been reported in many African settings; patients in this context are more likely to trust family members than medical providers (Breslin, 2005). In some settings, patients therefore seek out family members to safeguard against dominance by health providers (Ho, 2008). This perhaps explains why medical providers who were also family members were more trusted. However, there have been concerns that involvement of family members may compromise a patient's autonomy since they may have different values and priorities than those of the patient (Walter & Ross, 2014).

Interestingly, it was less the partners and more the health providers who were the source of strong paternalism in women's decisions in our study. Women reported that health providers were rarely questioned, as they were considered as experts who should be ‘obeyed’, especially when they had the dual role as a relative. Since we were considering these decisions in the context of implementation research, this raises ethical questions about women feeling free to question a doctor's advice or seek other opinions. Even within the shared decision-making model of clinical consent, it is important to strive for an equitable health provider–patient relationship free of coercion where health providers cannot use their influence to persuade women to participate in clinical care if they are unwilling. Whether research or clinical decisions, the strong influence of health providers and women's near-total deference to their recommendations indicates a need to sensitize health providers on the importance of engaging women's active involvement in shared decision making, especially during pregnancy when they are often subject to multiple inputs from others who feel they know best.

It could be argued that the strong social influences on women's decisions may not necessarily represent inappropriate persuasion but rather a sign of the workings of trust relationships. In a situation of profound uncertainty regarding a high-stakes decision, as in the case of new interventions during pregnancy, women may look to trusted others to guide them. In relationships of trust, many of us often willingly defer to the advice of others and do so without feeling that our autonomy has somehow been undermined. Delegating to others based on trust can be autonomy preserving (Gerhards et al., 2017). In contrast, some patients have reported that they did not want to be made to participate in shared decision making especially when the decision is scary and may wish to hold sole responsibility (Woolf et al., 2005).

Against the broader sociocultural backdrop in Kenya, women typically seek social support for important decisions during pregnancy from partners, friends, and family. This is arguably not unique to Kenya, and has been recently reported in a study conducted in Malawi (Sullivan et al., 2020), suggesting the value in appealing to models of ethical decision making during pregnancy, which allows for a role for others while recognizing women's agency to make important decisions affecting their health and health of their baby. Our data show why it remains difficult to disentangle the complex network of influences on women's decisions that are not always entirely benign. While, in principle, social support need not compromise a woman's autonomy when autonomy is viewed as deeply relational (Burkitt, 2016; Mackenzie & Stoljar, 2000), it is important that health providers and researchers attempt to distinguish willingly sought social support from the view held by some men that women must always obtain a man's consent because they do not have the right to consent on their own behalf.

There were several limitations to this study. Because our research ethics case study was linked with an ongoing PrEP demonstration study and took place before PrEP was widely available, this analysis primarily focused on social influences of taking PrEP during pregnancy because that was the central example under discussion with participants. However, the discussion guides included probes related to ‘new treatments’ more generally, and comparative examples of malaria and TB treatment during pregnancy. As mentioned, there were no notable differences in attitudes regarding partner involvement or other social influences across these examples, but the study was not designed to do a careful comparison across different interventions, rather to understand experiences in the context of PrEP. Further, because the primary research questions were not focused on the role of specific others in women's decision making, the data reported here emerged in a secondary analysis and therefore we lacked an opportunity to probe and follow up on the deeper dynamics of social influences. All men in our study were HIV infected, so had a motivation to have their partner participate in an HIV prevention trial centered around this idea of PrEP as a means of keeping a partnership together. They might have been more motivated or accepting of having their partners participate since they had an interest in having their partner take PrEP. We also cannot rule out the effects of peer influence in the male partner focus groups. Because we did not conduct separate individual interviews with men, it is possible that the views expressed were influenced by having other men in the room. We did have a Kenyan male facilitator for those group discussions, but we cannot rule out some posturing in those group discussions. Since July 2016 when the data were collected, we continue to see increased calls for the importance of including pregnant women in clinical research but there was a default practice of exclusion in earlier clinical trial phases. As subsequent studies have reported that PrEP is safe in pregnancy, there have been calls to more systematically include pregnant women in the scale-up of PrEP, as well as calls for more targeted research on safety and efficacy during pregnancy to address remaining critical gaps in understanding (Joseph Davey et al., 2020).

## Conclusions

Underinclusion of pregnant women in research continues to be a significant ethical barrier to the development of effective and safe interventions for use during pregnancy. Our study illustrates the importance of better understanding the roles not of only male partners, but also older female family members and clinicians, as potential barriers or advocates for pregnant women to use PrEP or other new medical interventions during pregnancy. Our findings confirm the multifarious nature of social influence on women's medical decision making during pregnancy, particularly with a new intervention when evidence is still emerging. This study identified a need for a model of ethical decision making and consent that better distinguishes beneficial and wanted social support from unwanted and potentially coercive influences on women's choices about their health. The study also points to the value of engaging health providers and family with emerging, relevant information about new interventions that may benefit women during pregnancy.

### Best Practices

Ethical guidance on the inclusion of pregnant women in research needs to be considerate and culturally sensitive to women's choices in contexts where the sociocultural norms allow for support. This should distinguish between mutually valued social support and the undesirable paternalistic norms that view women as unable to make decisions regarding their health and health of their baby during pregnancy. Additionally, ethical decision making should allow recognizing women's agency to make important decisions affecting their health and body once they become pregnant.

### Research Agenda

Our study has identified a need for primary research focusing on social influences in women's decision making, to participate in research during pregnancy that probe in-depth on social influences including from men who only participated in FGDs in this study. This will inform the development of a culturally sensitive model of ethical decision making and consent that better distinguishes beneficial and wanted social support from unwanted and potentially coercive influences on women's choices about their health and the health of their unborn baby during pregnancy.

### Educational Implications

There is a need to sensitize health providers on the importance of empowering women to engage actively in shared decision making in both clinical and research settings, especially during pregnancy when women reported that they would defer to influential others especially health providers and male partners. This will create an environment free of coercion and protect women from participating in research or clinical care against their wishes thereby safeguarding their autonomy.

## Supplemental Material

sj-docx-1-jre-10.1177_15562646211012296 – Supplemental material for Perceived Social Influences on Women's Decisions to use Medications not Studied in Pregnancy. A Qualitative Ethical Analysis of Preexposure Prophylaxis Implementation Research in Kenya Click here for additional data file. Supplemental material, sj-pdf-1-eeg-10.1177_15562646211012296 for Perceived Social Influences on Women's Decisions to use Medications not Studied in Pregnancy. A Qualitative Ethical Analysis of Preexposure Prophylaxis Implementation Research in Kenya by Kenneth Ngure, Susan B. Trinidad, Kristin Beima-Sofie, John Kinuthia, Daniel Matemo, Grace Kimemia, Anne Njoroge, Lillian Achiro, Jillian Pintye, Nelly R. Mugo, Elizabeth A. Bukusi, Jared M. Baeten, Renee Heffron, Grace John-Stewart and Maureen C. Kelley in Journal of Empirical Research on Human Research Ethics
