# Supplementary material for: Perceived Social Influences on Women's Decisions to use Medications not Studied in Pregnancy. A Qualitative Ethical Analysis of Preexposure Prophylaxis Implementation Research in Kenya
Source: J Empir Res Hum Res Ethics. 2021 Jun 16;16(3):225–37. doi: 10.1177/15562646211012296 (PMC8261769; doi:10.1177/15562646211012296)
Supplement: sj-docx-1-jre-10.1177_15562646211012296 – Supplemental material for Perceived Social Influences on Women's Decisions to use Medications not Studied in Pregnancy. A Qualitative Ethical Analysis of Preexposure Prophylaxis Implementation Research in Kenya [file sj-docx-1-jre-10.1177_15562646211012296.docx]

**Supplemental materials- Interview guides**

**CHIP (Choices in Pregnancy) – Demonstration Project**

**Focus Group Guide: Male Partners**

**I. Welcome & Introductions**

To get started, I’d like to invite everyone to get to know each other a bit. Could we go around the room and do brief introductions? If you feel comfortable, you could share –

- Your first name
- How many children you have?
- What made you decide to come to today’s discussion

Thank you. It is a pleasure to meet all of you.

**II. Deciding about Treatment**

To begin, I’d like us to talk about how people make decisions about whether to take medicine to prevent sickness.

**1. Scenario 1 (Man – Malaria):** So, first, let’s imagine a man—maybe a friend of yours, who is generally healthy. He tells you about his doctor offering him medication to **prevent malaria** and he wants your opinion on what he should do. Your friend’s doctor told him that this new drug has been tested by researchers and it is known to work well to prevent malaria. Your friend has some time to think about this before his next visit and asks your opinion.

- 1. Should your friend take this medication to prevent malaria? Why or why not?
  2. What kinds of information do you think your friend should know to make the decision whether to take the medicine or not? What questions do you think he should ask about the medication before deciding to take it or not?
  3. Do you think it would be okay for your friend to make this medical decision on his own, without seeking advice from his friends or family? Why or why not?

**2. Scenario 2 (Woman Malaria):** Okay, thanks. Now imagine your friend or relative **is a woman**. Same situation. She is offered a new medication to prevent malaria and has some time to think about the decision and asks your advice. What advice would you give her about this decision?

What kinds of information do you think she would need to make the decision whether to take the medicine or not?

1. Should your friend take this medication to prevent malaria? Why or why not?
2. What kinds of information do you think she should know to make the decision whether to take the medicine or not? What questions do you think she should ask about the medication before deciding to take it or not?
3. Do you think it would be okay for her to make this medical decision on her own, without seeking advice from his friends or family? Why or why not?
4. Are there certain considerations that only women need to ask about when considering medications?
5. Would your advice be different if this was not just a woman friend but was your wife? If so, how so?
6. Do you think it would be okay for a wife to make such a decision without consulting a husband? Why or why not?

**3.** **Scenario 3 (Women or Man HIV):** Now imagine your friend or relative—this time it could be a man or woman—is offered a new treatment to prevent **HIV**. It has been tested and shown to be effective in preventing HIV.

1. Should your friend take this medication to prevent HIV? Why or why not?
2. What kinds of information do you think he or she should know or ask about to make the decision whether to take the medicine or not?
3. Do you think the decision process is different for men and women in this situation involving HIV instead of malaria? If so, in what way?
   - - Prompt: For example, are there certain considerations that only women need to ask about when considering HIV medications?
4. Considering this in the context of HIV medication, do you think it would be okay for a male friend to make a decision on his own about taking the medication, without talking with his partner/wife? Why or why not?
5. Do you think it would be okay for a woman to make such a decision without consulting her husband? Why or why not?
6. In general, do you think that most people allow the doctor to make such decisions, or in your experience, do most people make such medical decisions with the doctor?

Now I’d like to ask your opinion about taking HIV prevention medications.

1. Do you think men, like yourselves, would prefer to use condoms or take a medication to prevent themselves from getting HIV?
   1. Could you explain your choice – why do you think men would prefer one over the other?

**III. Pregnancy Decisions**

Great, thank you for your thoughts. Next, I’d like us to talk about how couples make decisions about pregnancy and different choices made during the time a woman is pregnant.

1. If you wanted to have a baby with your partner, what would you do? (prompts: would you talk with her, ask her, tell her, etc).
2. If your partner wants to become pregnant, is this something she would discuss with you?
3. Did your partner become pregnant while you were participating in this study?
   1. If so, how was the choice made for her to become pregnant?
   2. If not, was there any reason why you chose not to have her become pregnant during this time?

Next, I am interested to know your opinions about a woman using medical treatments during her pregnancy. Some medical treatments could help the health of a woman, but we don’t know how they might affect the health of the baby. This is a difficult challenge for doctors and patients – how to decide whether to use a medication that will help a pregnant woman but may risk some harm to the unborn baby. So, I want to ask you about this.

1. If your partner wanted to take a medication to prevent her own illness while she was pregnant, what would you think about this?
   1. What concerns or questions would you have?
   2. What kinds of information would you like to have from the doctors?
2. Would you expect her to talk with you about the decision first, before deciding with her doctor?

**IV. HIV Prevention Research During Pregnancy**

So far, we have been talking about medications that have been proven effective in earlier research trials and are now ready for use by physicians. Other medications still need further research to learn how well they work and what side effects they might have for different people. One of the challenges is how to learn how medications work for women who are pregnant. The body changes during pregnancy and so drugs may work differently. Also, research is needed to find out about the safety for the woman and the unborn baby. So, there is some debate about whether it is ethically acceptable to do research with pregnant women.

1. In general, do you think pregnant women should be allowed to participate in research studies aimed at learning more about whether drugs are safe and effective during pregnancy? Why or why not? Under what circumstances do you think it might be okay, or do you think it is never okay?
2. Thinking specifically about HIV, some people believe that pregnant women should **not** be allowed to participate in HIV prevention studies because of the potential for harm to the baby. Other people believe that this kind of research is needed to help protect women from becoming infected with HIV and protect their babies from getting HIV – and that pregnant women who want to participate should be allowed to do so, even if there is some unknown risk to the baby.

What do you think about this?

- 1. Should pregnant women be allowed to participate in this kind of research (if they want to)?
  2. If your answer is “it depends,” or “in certain circumstances,” what considerations make a difference?
  3. Does HIV prevention research seem different to you from other kinds of prevention research with pregnant women – for example, research on medications to prevent malaria or TB? If so, what are those differences?

1. If you think it is sometimes or usually okay for a woman to participate in research while pregnant, do you think she must always get the permission of her partner, or does it depend?
2. If you could share your opinion with the people writing the rules about pregnant women and HIV research, what would you tell them? What kinds of considerations should they be thinking about?

**Closing and Wrap-Up**

Thank you. This has been very helpful, and we’ve reached the end of the questions I had for today.

Do you have any other questions or comments for us, or other issues you have not had a chance to mention?

Again, we know you are very busy with your lives and families. Thank you so much for sharing your ideas with us today. We hope this will help to improve how health providers and researchers talk with families about these decisions and help support them. Thank you very much.

If you think of anything else, you would like to say we will give you all a way to contact us with further comments. Have a lovely day and safe travels.

**CHIP (Choices in Pregnancy) – Stakeholders**

**Site: Ahero/Mathare**

**Focus Group Guide: Women**

**I. Introductions & Using Medications during Pregnancy**

To begin … ***[please use appropriate introduction re the color aliases]***

1. How do you think most people in your community think about using medication during pregnancy?

***PROBES, if needed:***

Safety/risk

Side effects

Efficacy

Social – family or community views

1. All of you are mothers, or are soon to be mothers. Please tell me a little about whether you think differently about using a medicine while you’re pregnant compared to when you’re not pregnant.
2. When a doctor prescribes a medication, what information is most important for women to know?

***PROBES, if needed:***

What it’s for

How it works

Dosing – how much, how often, for how long

Safety/risk

Side effects

Efficacy

Effects on baby

Cost

1. When a doctor prescribes a new medication, are women likely to ask a healthcare worker questions about the medication?
   1. ***If not:*** Why not?
   2. ***If so:***  What do they tend to ask?
   3. Are women likely to ask questions of anyone else? ***[If yes: tell me about that]]***
2. When a woman has been prescribed a new medication, who might she tell, and why?

***PROBES, if needed:***

Husband/partner

Friend

Relative

Doctor, nurse counselor, nurse

Mentor mother

Other

1. Whose opinions might shape a woman’s decision about whether to use a certain medication while she is pregnant?

***PROBES, if needed:***

Husband/partner

Friend

Relative

Doctor

Pharmacist

Nurse/nurse counselor

Mentor mother

Community/village members

Community leaders

Other

1. Why do you think women might decide not to take a medication during pregnancy?

***PROBES, if needed:***

Side effects to self

Inconvenience

Risk to baby

Stigma

Don’t believe medication is needed

Don’t believe medication will work

Others (eg, partner) don’t support medication use

1. Does the condition for which the medication is prescribed make a difference? For example, if the medication is to treat a malaria attack, vs. to treat something more serious, like HIV?
2. Do you think women are more likely to take a medication to treat an illness they already have or to take a medication to prevent them from getting an illness?
3. How do people feel about women using medications while breastfeeding? Any different concerns, compared with pregnancy?

**II. Risk-Benefit / Mother and Baby**

1. Do you think most pregnant women worry about the possible effects of medication on their unborn baby?
   1. ***If not:*** Why not?
   2. ***If so:*** What kinds of things are most concerning?
2. Let’s imagine that there are two medications available to treat a condition in a pregnant woman. Medication A is very effective in treating the problem in the woman, but we do not know how it might affect her baby. Medication B is less effective for treating the woman, but it is known to be safe for the baby.
   1. Which medication do you think a woman should take?
   2. Why?
   3. Who should make this decision?

***PROBES, if needed:***

Doctor/nurse

Woman

Partner

Woman with partner

Other

1. Today, pregnant women who are HIV-positive are given medication to protect the baby from getting HIV. Now imagine that the same medications could be used in HIV-negative women during pregnancy to prevent women from getting HIV.
   1. What do you think of this?
   2. Would women want to take this medication?
   3. Why or why not?
   4. What challenges might there be for women taking this medication?
   5. Who do you think should be offered this medication?

**III. HIV Prevention**

Researchers are working to find ways to protect women from becoming infected with HIV.

1. Do you think pregnant women (or breastfeeding women) in this area are at risk of getting HIV?
   1. If yes, which women are at risk?
   2. In your opinion, how can a woman know she is at risk for getting HIV?
2. If a woman is at risk of getting HIV, do you think she would prefer abstinence, using condoms, or taking pills during pregnancy to prevent getting HIV? (Why?)

**IV. HIV Prevention Research**

Because researchers are still learning about how the use of HIV medications during pregnancy might affect the baby, not very much research has involved pregnant women. Research on medications for HIV prevention has typically involved women who are not pregnant.

1. Do you think that HIV prevention research on new medications should include pregnant women?
   1. ***If not:*** Why not?
   2. ***If so:*** Why?
2. Imagine that your friend or sister is pregnant, and she has been invited to participate in a research study about a medication to prevent HIV. She asks your advice.
   1. What would you say?
   2. What questions – if any – would you have about the study?
   3. Would you encourage her to discuss this with anyone else (e.g., husband/partner, friends, elders)?
   4. Would you encourage or discourage her participation in the study? Why?
3. If you could share your opinion with the people writing the rules about pregnant women and HIV research, what would you tell them?
   1. Should pregnant women be invited to participate in this kind of research?
   2. Does HIV prevention research seem different to you from other kinds of prevention research with pregnant women (eg, malaria, TB)?

**V. Closing and Wrap-Up**

Thank you very much. This has been very helpful. We’ve reached the end of the questions I had for today.

1. Do you have any other comments or questions, or anything else you think we should know?

We know you are very busy with your lives and families. Thank you for making time to participate in this discussion.

**CHIP (Choices in Pregnancy) – Demonstration Project**

**Individual Interview Guide: Healthcare Workers**

**I. Treating Pregnant and Breastfeeding Women**

To get started, I’d like to ask generally about your experience as a healthcare worker taking care of pregnant women.

1. When you are treating a woman who is pregnant are you thinking mostly about the woman’s health, mostly about the unborn baby, or about both? Follow-up: In general, when caring for pregnant patients, how to you manage the balance between the mother and baby? Does it depend on certain factors?
2. More specifically, what questions or factors do you consider when deciding whether to give medication to a pregnant patient?
   1. Can you tell me an example of a medication where you would say, “I would *almost always* feel comfortable prescribing this to a pregnant patient?” What makes you come to that judgment?
   2. Can you tell me an example of a medication where you would say, “I would *never* feel comfortable prescribing this to a pregnant patient?” What makes you come to that judgment?
   3. What about an example where you would think, “It depends -- I *might* feel comfortable, but only in certain circumstances?” Can you describe a situation like that?
3. Do you think you would make a different decision prescribing a medication if the medication is being used to treat a condition the woman already has versus prescribing a medication to prevent a possible future health problem—say for example the woman has malaria and you prescribe a treatment for malaria, versus she doesn’t have malaria but lives in an endemic area and you are deciding whether to prescribe malaria prophylaxis?
4. Can you tell me how you think about treatment decisions for your pregnant patients in each of these scenarios—I am interested in what factors you consider, risks-benefits (interviewer, pause after each one to give time to respond): (1) for a pregnant woman who has syphilis, (2) a pregnant woman who has active TB, (3) a pregnant woman who has malaria, (4) and a pregnant woman who is healthy but whose partner is known to be HIV+.
5. Thinking about these same factors in your clinical decision making, can you discuss how you make such decisions for a woman who is breastfeeding?
   1. What questions or factors do you consider during this period?
   2. On the spectrum, do you think about breastfeeding women as being more like your non-pregnant patients or more like your pregnant patients?
6. When you are talking with female patients about taking a medication during pregnancy or breastfeeding….
   1. What information do you think it is important to share with pregnant patients about a medication you are recommending?
   2. Do pregnant or breastfeeding women tend to have questions about the effects of medication on their child?
   3. Is it your impression that women are expected to discuss this kind of decision with their partners? With other people?
   4. Do you ever have women ask your advice without consulting their partner/husband? How do you handle such situations?
7. Thank you. Now let’s think for a moment about the research setting. In research, the goal is to advance scientific knowledge, rather than to focus exclusively on the treatment and well-being of the patient. Are there different issues that you think would come up in including pregnant or breastfeeding women in research, compared to the clinical context, which we’ve already discussed?
8. If one of your patients had the chance to enroll in a study on a new drug to prevent malaria or TB, but she is pregnant, what advice would you give her on whether to participate in the research?

**II. Specific to PrEP**

Thank you. Now I’d like to ask you a few questions about issues that might come up when considering the use of PrEP during pregnancy and breastfeeding.

1. Since working on this study, what have been your experiences providing PrEP to pregnant women?
   1. How many women have you taken care of who became pregnant while taking PrEP?
   2. Did they talk with you about the decision to become pregnant before becoming pregnant? After becoming pregnant.
   3. Is it your experience that the pregnancies are planned by the women? In discussion with her partner?
   4. What types of concerns did they have about PrEP, if any, before becoming pregnant?
   5. After becoming pregnant.
2. What do you think about the use of PrEP to prevent HIV in pregnant women?
   1. What challenges, if any, have you had providing PrEP to pregnant women?
   2. Based on your experiences within this research study, when you think about moving PrEP into routine clinical practice in Kenya, what types of challenges do you think there might be?
3. In thinking about caring for your pregnant patients outside of this research study, what information would help you decide whether to offer PrEP?
4. What kind of evidence would you consider most important/useful in your decision making?
   - - Risk or benefit of treatment to the woman
     - Risk or benefit of treatment to the baby
     - Risk of acquiring HIV for the woman (and baby)
     - Guidelines recommending that you offer PrEP to pregnant women
5. If evidence or guidelines were available about the use of HIV prevention medications in pregnant and breastfeeding women, what sources would you consider to be most trustworthy?
6. Do you think about HIV interventions differently if they treat the mother in pregnancy/postpartum to prevent the infant from acquiring HIV or prevent the mother from getting HIV during pregnancy or breastfeeding? What issues come to mind in these two scenarios?

**Closing and Wrap-Up**

Thank you. This has been very helpful, and we’ve reached the end of the questions I had for you today.

1. Do you have any other questions or comments for us, or other issues you have not had a chance to mention?

Again, we know you are very busy with your lives and families. Thank you so much for sharing your ideas with us today. We hope this will help to improve how health providers and researchers talk with women about these decisions and help support them. Thank you very much.

If you think of anything else, you would like to say we will give you all a way to contact us with further comments. Have a lovely day and safe travels.

**CHIP (Choices in Pregnancy) - Demonstration Project**

**Individual Interview Guide: Women**

**I. Introduction**

To get started, it would help if you could tell me a bit about your experience with the Partner’s Demonstration Project.

1. Thinking back, how did you learn about the research study, in the first place?
2. What made you want to find out more about it?
3. What made you decide to participate in the research study? [Probes, if needed: avoid HIV for self; avoid HIV for child; free or low-cost medical care; help researchers/future patients; social pressure; incentive; other]
4. Did you sign up with anyone you know? A friend or relative?
5. Did you talk with anyone in your life – perhaps a family member, partner, friend, or other trusted person – about whether to participate in the research?
   1. If yes: What did, they have to say? Did you take their advice? Why or why not?
6. Do you remember talking with a member of the study staff when you were deciding whether to join the study?
7. Can you tell me the main things you talked about, or were told, in that conversation?
   1. What seemed most important to you at the time?
   2. Did you have any questions?
   3. Did you have any questions that occurred to you after you started? Did you ask these questions and if so, were they answered to your satisfaction? Did you feel comfortable to ask questions at any time?
   4. Were there things that you wished someone had explained to you more at the beginning of your participation?
8. When thinking about preventing HIV, do you think a woman, similar to yourself, would prefer to use condoms or take a medication?
   1. Could you explain your reasons —what is better about one over the other?)

**II. HIV Prevention vs. Other Choices**

Thank you. One of the things we are trying to learn about is whether taking medicine to prevent HIV is different from other health decisions a woman might make during pregnancy. In this part of the interview, I will ask you about your choice to become pregnant while taking PrEP.

1. How did you decide to become pregnant while taking PrEP? Clarification if needed: Was the pregnancy planned or did it just happen unexpectedly?
2. If it was planned, did you talk about the choice to become pregnant while taking PrEP with anyone before becoming pregnant (partner, friend or relative, healthcare provider)?
3. What questions or concerns did you have?
4. What types of information did people share with you that was helpful?
5. What about after you became pregnant—did you talk with anyone (partner, friend or relative, health provider) about staying on PrEP during your pregnancy?
   1. What questions or concerns did you have?
   2. What types of information did people share with you that was helpful?
6. Did you decide to continue or stop using PrEP while you were pregnant? How did you make that choice—what types of things did you consider or think about? For example, related to your own health or that of your baby? [Interviewer note: Be sure to include the above prompt about women’s own health and health of baby. Then give the women plenty of time to think about this open question first, and to respond, before proceeding to specific questions below. Pause after each to give time for her consideration and responses.]
   1. Did you talk about your decision with your partner? Healthcare worker?
   2. What information was the most helpful to you in making that choice?
   3. What information was the least helpful to you in making that choice?
   4. What other information or support would you like to have had?
   5. Would you make the same choice if you had to make the decision again today?
   6. In light of your experience, what advice would you give to another woman who faces the same choice—who is on PrEP to prevent HIV and who becomes pregnant?
7. If you had a sister or good friend who was taking PrEP and wanted to also become pregnant, or became pregnant, what would your advice be to her about whether to stay on the medication?
8. If you could share your opinion with the people writing the rules about including or not including pregnant women in HIV research, what would you tell them? What should they be thinking about from your perspective?
   1. Should pregnant women be allowed to participate in this kind of research (if they want to)?
   2. If your answer is “it depends,” or “in certain circumstances,” what considerations make a difference?
   3. Does HIV prevention research seem different to you from other kinds of prevention research with pregnant women – for example, malaria or TB? If so, how is it different with other diseases?

**III. Policy Questions**

There is discussion among researchers and those responsible for making sure research is done in a good way about whether research in pregnant women is ethically acceptable. Some people believe that pregnant women should not be allowed to participate in research studies on because there might be risks for the baby. Other people believe that this kind of research is needed to see if there is benefit to the mother and/or baby. So, many people disagree about this important issue.

1. What do you think about this? Should women who want to participate in research studies while they are pregnant be allowed to do so? Why or why not?

**Closing and Wrap-Up**

Thank you. This has been very helpful, and we’ve reached the end of the questions I had for you today.

1. Do you have any other questions or comments for us, or other issues you have not had a chance to mention?

Again, we know you are very busy with your lives and families. Thank you so much for sharing your ideas with us today. We hope this will help to improve how health providers and researchers talk with women about these decisions and help support them. Thank you very much.

If you think of anything else, you would like to say we will give you all a way to contact us with further comments. Have a lovely day and safe travels.
